# Potential Prognostic and Diagnostic Application of a Novel Monoclonal Antibody Against Keratinocyte Growth Factor Receptor

**DOI:** 10.1007/s12033-014-9773-x

**Published:** 2014-06-05

**Authors:** Simona Ceccarelli, Roberto Bei, Enrica Vescarelli, Sirio D’Amici, Cira di Gioia, Andrea Modesti, Ferdinando Romano, Adriano Redler, Cinzia Marchese, Antonio Angeloni

**Affiliations:** 1 Department of Experimental Medicine, Sapienza University of Rome, Viale Regina Elena 324, 00161 Rome, Italy; 2 Department of Clinical Sciences and Translational Medicine, University of Rome “Tor Vergata”, Via Montpellier 1, 00133 Rome, Italy; 3 Department of Radiological Sciences, Oncology and Anatomical Pathology, Sapienza University of Rome, Viale Regina Elena 324, 00161 Rome, Italy; 4 Department of Public Health and Infectious Diseases, Sapienza University of Rome, Piazzale Aldo Moro 5, 00185 Rome, Italy; 5 Department of Surgical Sciences, Sapienza University of Rome, Viale Regina Elena 324, 00161 Rome, Italy; 6 Department of Molecular Medicine, Sapienza University of Rome, Viale Regina Elena 324, 00161 Rome, Italy

**Keywords:** Keratinocyte growth factor receptor, Monoclonal antibody, Breast cancer, Pancreatic adenocarcinoma, Papillary thyroid carcinoma

## Abstract

KGFR is involved in the pathogenesis of several human cancers. In this study, we generated and characterized a monoclonal antibody specific to KGFR (SC-101 mAb) and evaluated its potential use in basic research and as a diagnostic and prognostic tool. The specificity and biological activity of the SC-101 mAb were evaluated by Western blotting, immunofluorescence, and immunoprecipitation analyses on various cell lines. KGFR expression in breast, pancreatic, and thyroid carcinoma was assessed by immunohistochemistry (IHC) with SC-101 mAb. KGFR expression levels revealed by SC-101 mAb resulted to increase proportionally with tumor grade in breast and pancreatic cancer. In addition, SC-101 mAb was able to detect KGFR down-modulation in thyroid cancer. SC-101 mAb might represent a useful tool for basic research applications, and it could also contribute to improve the accuracy of diagnosis and prognosis of epithelial tumors.

**Electronic supplementary material:**

The online version of this article (doi:10.1007/s12033-014-9773-x) contains supplementary material, which is available to authorized users.

## Introduction

Fibroblast growth factor receptors (FGFRs) family consists of four highly related genes, FGFR1-4, encoding proteins that are 55–72 % identical in their amino acid sequence. This family is characterized by a complexity of heterodimers and a high frequency of alternative splicing events, which justifies the signal transduction of a large number of ligands [1]. In particular, FGFR2 gene is subjected to an alternative splicing that generates two isoforms, the Keratinocyte growth factor (KGFR or FGFR2-IIIb) and the FGFR2-IIIc. The domains designated IIIb and IIIc, located on the third Ig loop of the FGFR2, are encoded by alternative usage of exon 8 or 9 of the FGFR2 gene. This rearrangement is cell type-dependent, since KGFR is expressed predominantly on epithelial cells, while FGFR2-IIIc is detected in cells of mesenchymal lineages. The two isoforms differ also for ligand specificity, since KGFR isoform binds with high affinity to FGF7/KGF, FGF10 and FGF22, and with low affinity to FGF1 and FGF3, while FGFR2-IIIc isoform binds to FGF1, FGF2, FGF4, FGF5, FGF6, FGF8, FGF9, FGF16, FGF17, FGF18, FGF20, FGF21, and FGF23 [2].

Given the role of both FGFR2 isoforms in inducing cell proliferation, it has been observed that their altered expression can be associated to loss of proliferation control, as documented in various types of cancer. Nevertheless, it is still controversial whether these receptors should be considered oncogenes. Indeed, they not only act as positive regulators of tumorigenesis by stimulating cell growth, but they also possess tumor suppressor properties, by enhancing cell differentiation. In fact, overexpression of FGFR2 gene has been reported in breast, lung, stomach, and pancreatic cancers [3], while its down-modulation has been demonstrated in thyroid cancer [4] and in melanoma, where FGFR2 gene can even present loss-of-function mutations [5].

Moreover, the two FGFR2 isoforms can play different roles in tumorigenesis and their effects depend upon the cell type in which they are expressed. In particular, FGFR2-IIIc expression has been correlated to epithelial to mesenchymal (EMT) transition of tumor cells in bladder cancer, which may represent a key factor in tumor progression by increasing the metastatic potential of cancer cells [6]. As for KGFR, in some cell types, its overexpression can lead to mitogenesis and tumor progression [7], but in other context it can induce cell differentiation, thus reducing the invasive potential of tumor cells [8].

The evaluation of expression and specific contribution of the two isoforms in various types of cancer is complicated by the lack of commercial antibodies that are able to discriminate between KGFR and FGFR2-IIIc. In fact, the only way to selectively analyze the splicing variants is to make use of specific and expensive molecular biology techniques, such as Real-Time PCR.

In light of these considerations, the aim of this study was the generation and characterization of a monoclonal antibody specific for KGFR. We demonstrated that such novel antibody (SC-101 mAb) selectively recognizes this isoform. In addition, we observed that SC-101 mAb is particularly efficient in detecting KGFR expression in tumor specimens. Indeed, we were able to show a significant correlation between intensity of antibody staining in immunohistochemistry (IHC) and tumor grade. Thus, SC-101 mAb might represent a specific tool for KGFR identification to be used as a diagnostic and prognostic tool in different types of epithelial tumors.

## Materials and Methods

### Ethics Statement

All experiments with human samples were conducted according to the principles expressed in the Declaration of Helsinki and approved by the Ethics Committee of the Azienda Policlinico Umberto I of Rome (official name of the committee). Following the Institutional Guidelines, written informed consent was obtained from all patients prior to their inclusion in the study.

### Reagents and Cell Cultures

MCF-7, HEK293, and HeLa cells were obtained from the American Type Culture Collection (ATCC-LGC Promochem, Teddington, UK) and cultured in Dulbecco’s Modified Eagle’s Medium (DMEM; Invitrogen, Karlsruhe, Germany), supplemented with 10 % fetal bovine serum (FBS; Invitrogen) and antibiotics. Primary cultures of human fibroblasts (HF) were established from 1 cm^2^ full-thickness skin biopsy from a healthy donor, as previously described [9], and maintained in DMEM containing 10 % FBS.

### Production of the Recombinant Protein Corresponding to the Product of Exon 8 of the FGFR2 gene

Total RNA was extracted from MCF-7 cells using TRIzol (Invitrogen) in accordance with the manufacturer’s instructions. cDNA was synthesized by reverse transcription. The exon 8 sequence of FGFR2 gene was amplified from the cDNA pool by PCR with Platinum Taq DNA polymerase (Invitrogen) using the following primers:

Forward, 5′-GGGGGATCCCACTCGGGGATAAATAGTTCC-3′;

Reverse, 5′-GGGAAGCTTGCTGTTTTGGCAGGACAGT-3′.

The amplification products were purified using the Wizard^®^ SV Gel and PCR Clean-Up System (Promega, Madison, WI, USA), cloned into pET-30a plasmid carrying His-Tag at both N- and C-terminal (Novagen, Darmstadt, Germany), and subsequently transformed into *E. coli* BL21 (DE3) pLysS expression strain (Promega). Positive clones were sequence-verified. Then, recombinant protein expression was induced by 1 mM Isopropyl-beta-D-thiogalactoside (IPTG; Sigma-Aldrich, srl, Milano, Italy). The recombinant protein was purified by His-Bind Kits (Novagen) and quantified by BSA Protein Assay (Bio-Rad Laboratories srl, Segrate, MI, Italy).

### Production of SC-101 mAb

SC-101 mAb was generated using BALB/C mice immunized by intraperitoneal injections of the recombinant protein corresponding to the product of exon 8 of the FGFR2 gene. The myeloma cell line NS1 was used as a fusion partner, through the addition of 1 ml of polyethilenglycole (PEG). Hybridomas supernatants were tested for antibody-binding activity by ELISA and by indirect immunoperoxidase method on frozen sections of human skin. Selected hybridoma clones were expanded for ascites tumor production.

For large-scale antibody production, hybridoma cells were injected intraperitoneally in BALB/c mice. Ascites fluid was collected after 10–15 days, clarified by centrifugation, and stored at −20 °C. SC-101 mAb was purified from mice ascites by Montage antibody purification Kit with Prosep-G plug (Millipore, Billerica, MA, USA).

### Immunofluorescence Microscopy

MCF-7 and HeLa cells, grown on coverslips, treated or not with 100 ng/ml human recombinant KGF (Millipore) for 10 min, were processed for immunofluorescence as previously described [10] and incubated with SC-101 mAb (1:100 or 1:500 in PBS) or with a rabbit polyclonal antibody raised against the intracellular domain of FGFR2 (Bek C-17; 1:20 dilution; Santa Cruz Biotechnology, Santa Cruz, CA, USA). Primary antibodies were visualized using the appropriate FITC-conjugated IgG (1:100 in PBS; Jackson ImmunoResearch Laboratories, West Grove, PA, USA). Nuclei were visualized using 4′, 6-diamido-2-phenylindole dihydrochloride (DAPI) (1:10000 in PBS; Sigma-Aldrich). Positively stained area was measured with the aid of NIH ImageJ v1.56 (National Institutes of Health, Bethesda, MD). Staining intensity was scored on a scale of 0–3. The score 0 corresponds to totally negative cases. Score 1, 2, and 3 were assigned to weak, moderate, and strong staining, respectively. Final IF score for MCF-7 and HeLa cells was obtained by multiplying the staining intensity score by the percentage of positively KGFR-stained area. The final score classified the samples into four groups: negative, weak positive, moderate positive, and strong positive (Table 1).

**Table 1:** Assignment of immunofluorescence score according to staining intensity and percentage of positively stained area

Score	Positive area (%)	Intensity
0	0–10	Negative
1	11–17	Weak positive
2	18–25	Moderate positive
3	>25	Strong positive

### Western Blot and Immunoprecipitation Analysis

MCF-7, HeLa, HEK293, and HF cells were lysed in RIPA buffer. Total proteins (50–150 μg) were resolved under reducing conditions by 8 % SDS–PAGE and transferred to Immobilon-FL membranes (Millipore). Membranes were incubated for 1 h with blocking solution (Blotting Grade Blocker, Bio-Rad Laboratories Headquarters, Alfred Nobel Drive Hercules) and overnight at 4 °C with SC-101 mAb (1:1000) or with Bek C-17 polyclonal antibody (1:200), followed by goat anti-mouse or goat anti-rabbit horseradish peroxidase (HRP)-conjugated secondary antibody (Sigma-Aldrich). Bound antibody was detected by enhanced chemiluminescence detection reagents (Pierce Biotechnology Inc, Rockford, IL, USA), according to manufacturer’s instructions. MCF-7 cells pretreated with 3 μg/ml SC-101 antibody for 30 min were treated with 10 ng/ml human recombinant KGF for 10 min, lysed in RIPA buffer and subjected to Western blotting analysis with phosphor-ERK antibody (1:1000; Cell Signaling Technology, Danvers, MA, USA) and ERK2 antibody (1:200; Santa Cruz Biotechnology), followed by goat anti-mouse or goat anti-rabbit horseradish peroxidase (HRP)-conjugated secondary antibody (Sigma-Aldrich). For immunoprecipitation analysis, 1 mg of total protein was immunoprecipitated with 4 μg/ml SC-101 mAb or Bek C-17. Immunocomplexes, aggregated with 50 μl of γ-bind protein-G Sepharose (Amersham Biosciences, Uppsala, Sweden), were washed four times with 0.6 ml of buffer, resolved under reducing conditions by 8 % SDS–PAGE and transferred to membranes. Membranes were incubated with Bek C-17 polyclonal antibody (1:200) overnight at 4 °C followed by the appropriate HRP secondary antibody and enhanced chemiluminescence detection.

### Immunohistochemistry (IHC)

Immunohistochemistry was performed on breast and pancreatic cancer tissue microarrays (Us Biomax, Inc., USA). Breast tissue array included 40 cases of invasive ductal breast cancer (grade 2–3), and pancreas tissue array included 40 cases of duct adenocarcinoma, grade 1–3. Each array also contained 5 normal adjacent tissues (NAT) and 5 normal tissues, duplicate cores per case. IHC analysis was performed also on paraffin sections of thyroid cancer tissues and normal tissues obtained from 11 patients from the Department of Surgical Sciences (Sapienza University of Rome). Sections were dewaxed and rehydrated with xylene and decreasing concentrations of ethanol to water. After blocking of endogenous peroxidase activity, antigen retrieval was performed in preheated ethylenediamine tetraacetic acid (EDTA) buffer (pH 8) in a microwave for 15 min. Sections were stained using a 1:300 dilution of SC-101 mAb for 1 h at 25 °C. Binding of the primary antibody was checked by LSAB+ System HRP (DakoCytomation, Inc., Carpinteria, CA, USA), and 3, 3′-diaminobenzidine (DAB) as a chromogen. Slides were counterstained with hematoxylin, dehydrated, and mounted. Positively stained area was measured with the aid of NIH ImageJ v1.56 (National Institutes of Health, Bethesda, MD), as previously described [11]. Staining intensity was scored on a scale of 0–3. The score 0 was attained for totally negative cases. For weak, moderate, and strong staining, the scores were 1, 2, and 3, respectively. Final IHC score for each group of samples was obtained by multiplying the staining intensity score by the percentage of positively KGFR-stained area. The final score classified the samples into four groups: negative, weak positive, moderate positive, and strong positive (Table 2).

**Table 2:** Assignment of immunohistochemical score according to staining intensity and percentage of positively stained area

Score	Positive area (%)	Intensity
0	0–15	Negative
1	16–40	Weak positive
2	41–150	Moderate positive
3	>150	Strong positive

### Quantitative Real-Time PCR

Biopsies of breast, pancreatic, and thyroid carcinomas, and of the correspondent healthy tissues, were processed for total RNA extraction with the use of TRIzol reagent (Invitrogen). cDNA was generated using the SuperScript III Reverse Transcriptase Kit (Invitrogen). For KGFR and FGFR2-IIIc, specific custom TaqMan^®^ Primer/Probe assays were developed (Table 3). Assays were conducted in triplicate on an ABI 7500 Real-Time instrument (Applied Biosystems by Life Technologies, Carlsbad, CA, USA) using the following conditions: 50 °C for 2 min, 95 °C for 10 min, and then 95 °C for 15 s and 60 °C for 1 min, repeated 40 times. Relative quantification was performed using GAPDH mRNA or MT-ATP6 mRNA as endogenous control.

**Table 3:** Custom TaqMan Assay gene-specific primers and reporter probe

Gene	Forward primer sequence	Reverse primer sequence	Reporter sequence
KGFR	GGCTCTGTTCAATGTGACCGA	GTTGGCCTGCCCTATATAATTGGA	TTCCCCAGCATCCGCC
FGFR2-IIIc	CACGGACAAAGAGATTGAGGTTCT	CCGCCAAGCACGTATATTCC	CCAGCGTCCTCAAAAG

### Statistical Analysis

Each set of experiments was repeated at least in triplicate, and standard deviation values were calculated. Student’s two-tailed *t* test was used for statistical analysis, and *p* values less than 0.05 were considered statistically significant.

## Results

### SC-101 mAb Specificity

**Fig. 1:**
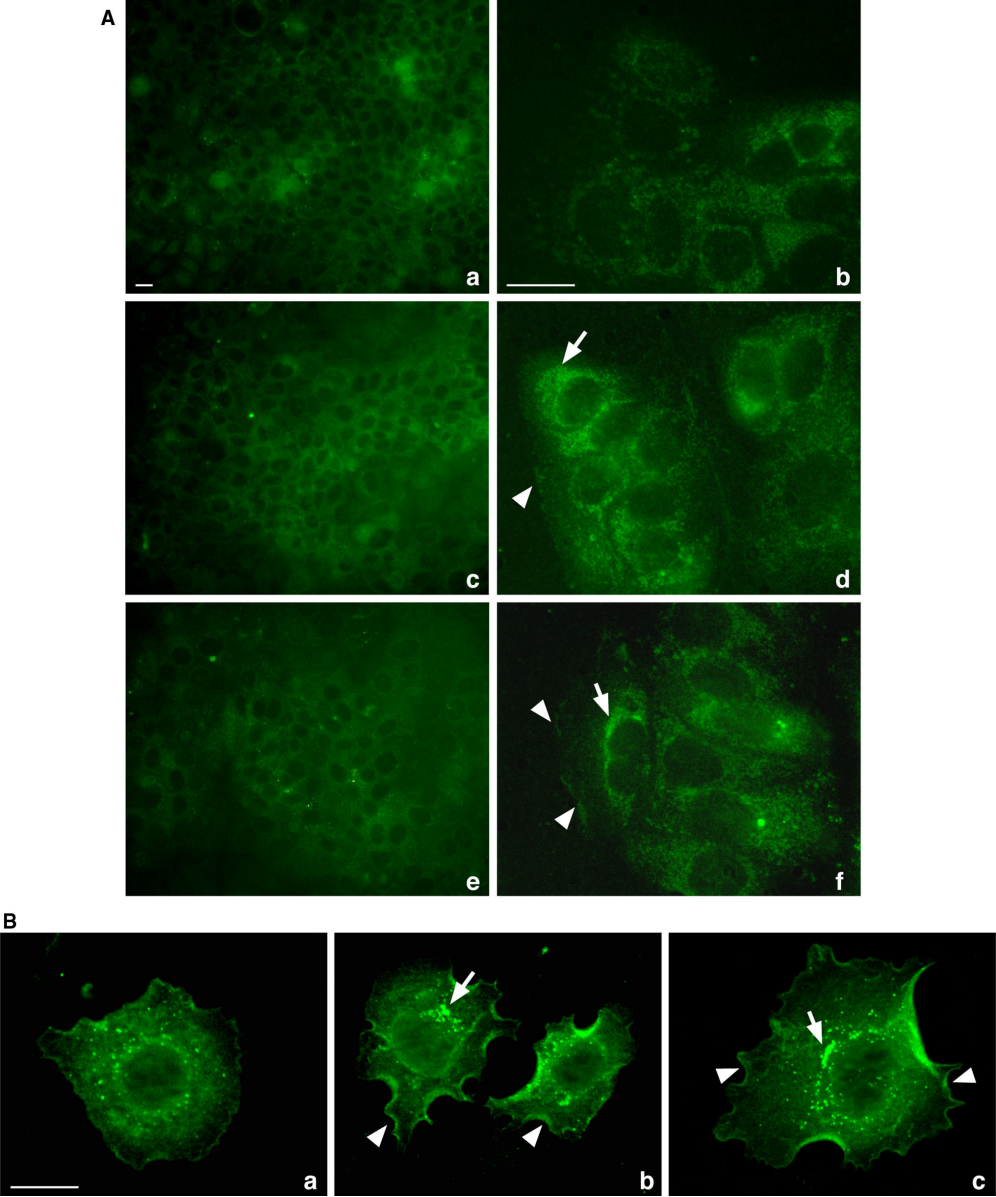
SC-101 mAb functionality in immunofluorescence analysis. MCF-7 cells (**A**) or HeLa cells (**B**) were subjected to immunofluorescence with a polyclonal antibody directed against FGFR2 (Bek C-17, 1:20) (**A**, panels *a, b* and **B**, panel *a*) and with two different dilutions of SC-101 mAb (1:100, **A,** panels *c, d* and **B,** panel *b*; 1:500, **A,** panels *e, f* and **B,** panel *c*) followed by the appropriate FITC-conjugated secondary antibody (*green*). KGFR signal was detected intracellularly (*arrows* in **A**, panels *d, f* and **B**, panels b, c) and on cell membrane (arrowheads in **A**, panels d, f and **B**, panels b, c). *Scale bars* 20 μm. Staining intensity was calculated for each cell lines. Briefly, we took three images from each cell lines and mean values were obtained from five measurements of each image and classified as scores according to Table 1 (Color figure online)

**Fig. 2:**
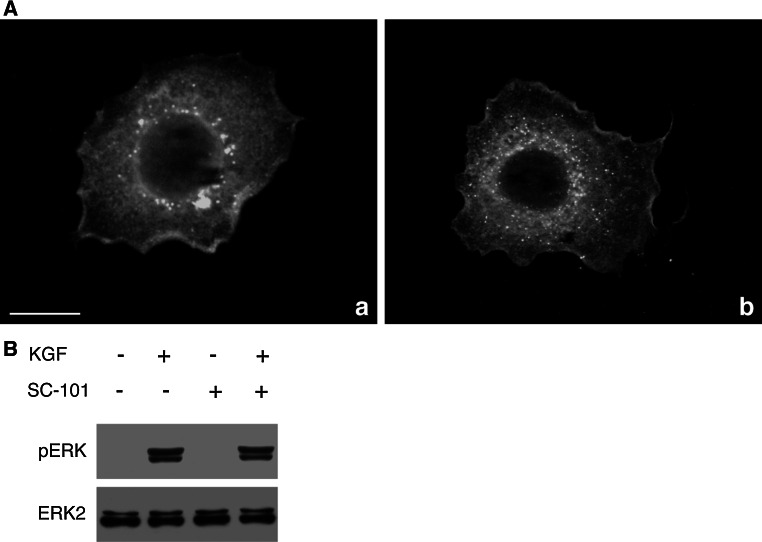
SC-101 mAb functionality in the presence of KGF. **A** HeLa cells untreated (panel *a*) or treated with 100 ng/ml human recombinant KGF for 10 min (panel *b*) were subjected to immunofluorescence with SC-101 mAb (1:100) followed by the appropriate FITC-conjugated secondary antibody (*green*). KGFR signal showed the same pattern even after KGF binding. *Scale bar* 20 μm. **B** Western blotting analysis of ERK phosphorylation status in MCF-7 cells treated or not with 10 ng/ml human recombinant KGF for 10 min, in the presence or absence of pretreatment with 3 μg/ml SC-101 for 30 min. ERK phosphorylation was evaluated by blotting with an anti-phospho-ERK antibody. Western blotting with anti-ERK2 antibody was used as loading control. The images are representative of at least three independent experiments (Color figure online)

To validate SC-101 mAb, immunofluorescence analysis was initially performed. With respect to the commercial Bek C-17 (Fig. 1A, panels a, b), widely used as standard antibody for FGFR2 evaluation, SC-101 mAb (Fig. 1A, panels c–f) resulted to be more efficient in detecting KGFR expression on cell membrane (arrowheads in Fig. 1A, panels d, f) and the intracellular signal likely due to KGFR protein synthesis (arrows in Fig. 1A, panels d, f) in MCF-7 cells. Quantification of IF signal in MCF-7 stained with SC-101 1:100 dilution (Table 4), revealed a final IF score of 30.4 ± 19.1 in MCF-7 cells which corresponded to a strong positive staining (score 3), according to our classification (Table 1). Moreover, SC-101 mAb showed a higher sensitivity, thus allowing us to obtain a strong signal even at lower concentrations (Fig. 1A, panels e, f) (23.6 ± 17.6, score 2). To better highlight KGFR membrane signal, we performed immunofluorescence analysis also on HeLa cells (Fig. 1B), which at low-density conditions occur as single cells rather than cell clusters as MCF-7 cells (Fig. 1A). Quantification of IF signal revealed a final IF score of 66 ± 25.6 in HeLa cells stained with SC-101 1:100 dilution (Table 4). This score corresponded to a strong positive staining (score 3), according to our classification (Table 1). In HeLa cells stained with SC-101 1:500 dilution, we measured an IF score of 42.3 ± 1.7 (score 3). Since the antigen for generating SC-101 antibody overlaps a region of the receptor that has been shown to bind KGF [12], we assessed if treatment with KGF could interfere with receptor-antibody binding. To this end, we performed immunofluorescence analysis with SC-101 antibody on HeLa cells untreated or treated with KGF. Figure 2A confirmed that SC-101 antibody was able to recognize KGFR also after binding with its ligand. Moreover, we also investigated whether SC-101 antibody binding to KGFR could determine receptor activation or could interfere with its KGF-mediated activation. Treatment with SC-101 antibody alone did not induce KGFR signal transduction, as indicated by absence of ERK phosphorylation, while KGF was still able to activate ERK kinases even after pretreatment with SC-101 antibody (Fig. 2B).

**Table 4:** Mean values of staining intensity, percentage of positively stained area and final immunofluorescence score in MCF-7 and HeLa cells

SC-101 1:100 dilution	Positive area (%)	Intensity	IHC score
MCF-7	19.4 ± 3.6	2 ± 0.8	30.4 ± 19.1
HeLa	26.0 ± 2.9	3 ± 0.7	66 ± 25.6

SC-101 1:500 dilution	Positive area (%)	Intensity	IHC score
MCF-7	14.6 ± 4.9	2 ± 0.7	23.6 ± 17.6
HeLa	21.15 ± 0.85	2 ± 0	42.3 ± 1.7

**Fig. 3:**
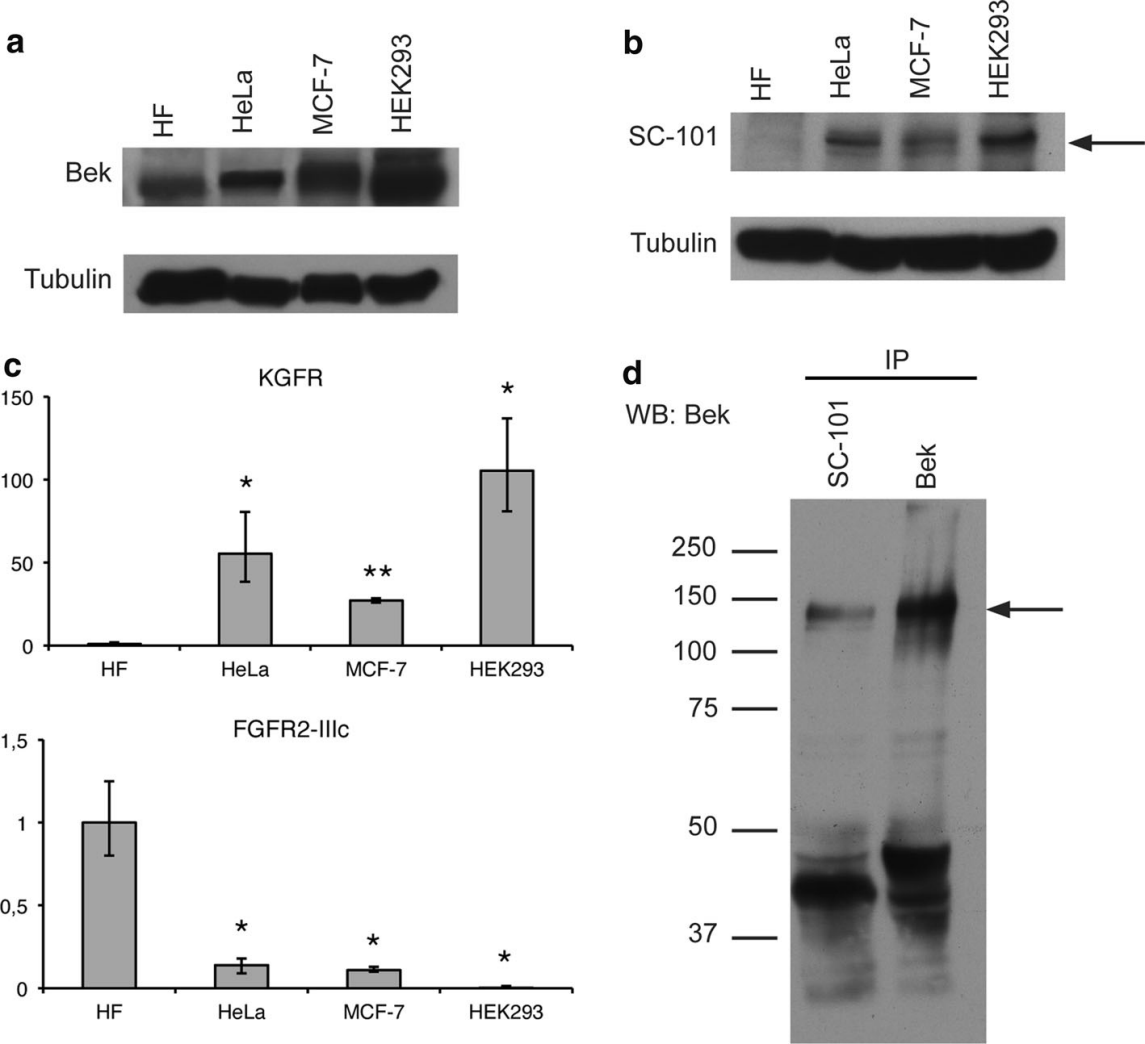
SC-101 mAb functionality and specificity. **a, b** Western blotting analysis of KGFR protein levels in HF, HeLa, MCF-7 and HEK293 cells. KGFR protein expression was evaluated by blotting with an anti-Bek antibody (**a**) or with SC-101 mAb (**b**). Western blotting with anti-Tubulin antibody was used as loading control. The images are representative of at least three independent experiments. KGFR band is indicated by the *black arrow*. **c** Quantitative Real-Time PCR analysis of KGFR and FGFR2-IIIc mRNA expression in HF, HeLa, MCF-7 and HEK293 cells. Relative KGFR or FGFR2-IIIc mRNA levels are shown as fold value of the level of the corresponding mRNA in HF cells. Error bars represent standard deviations. *p < 0.05, **p < 0.01. **d** Immunoprecipitation assay was performed to evaluate the SC-101 ability to bind the native form of KGFR. KGFR protein was immunoprecipitated with SC-101 mAb or with Bek C-17 antibody and blotted with Bek C-17 antibody. The images are representative of at least three independent experiments

To further characterize the specificity of SC-101 mAb, we performed Western blotting analysis on HeLa, MCF-7, and HEK293 cell lysates, which are known to express KGFR, and on HF cells, which express only the FGFR2-IIIc isoform. As shown in Fig. 3a, the commercial Bek antibody recognized in all cell lines a band with a molecular weight of 145 kDa, corresponding to KGFR/FGFR2-IIIc protein. Conversely, SC-101 mAb detected the same band, corresponding to KGFR protein, in HeLa, MCF-7, and HEK293 cell lines, while no apparent detection of endogenous FGFR2-IIIc in HF cell lysates was observed (Fig. 3b). To support protein findings, we also assessed KGFR and FGFR2-IIIc mRNA expression on the same cell lines. Figure 3c confirms that KGFR is consistently expressed in HeLa, MCF-7 and HEK293 cells, and not in HF cells. On the contrary, all the epithelial cell lines expressed negligible levels of FGFR2-IIIc compared to that of HF cells. Subsequently, we evaluated the ability of SC-101 mAb to immunoprecipitate KGFR protein. As shown in Fig. 3d, KGFR protein from MCF-7 cells extract was immunoprecipitated with SC-101 mAb or Bek antibody and detected by blotting with Bek antibody. The data obtained displayed that SC-101 mAb was able to immunoprecipitate the native form of KGFR.

### Immunohistochemical Detection of KGFR by SC-101 mAb in Breast Cancer

**Fig. 4:**
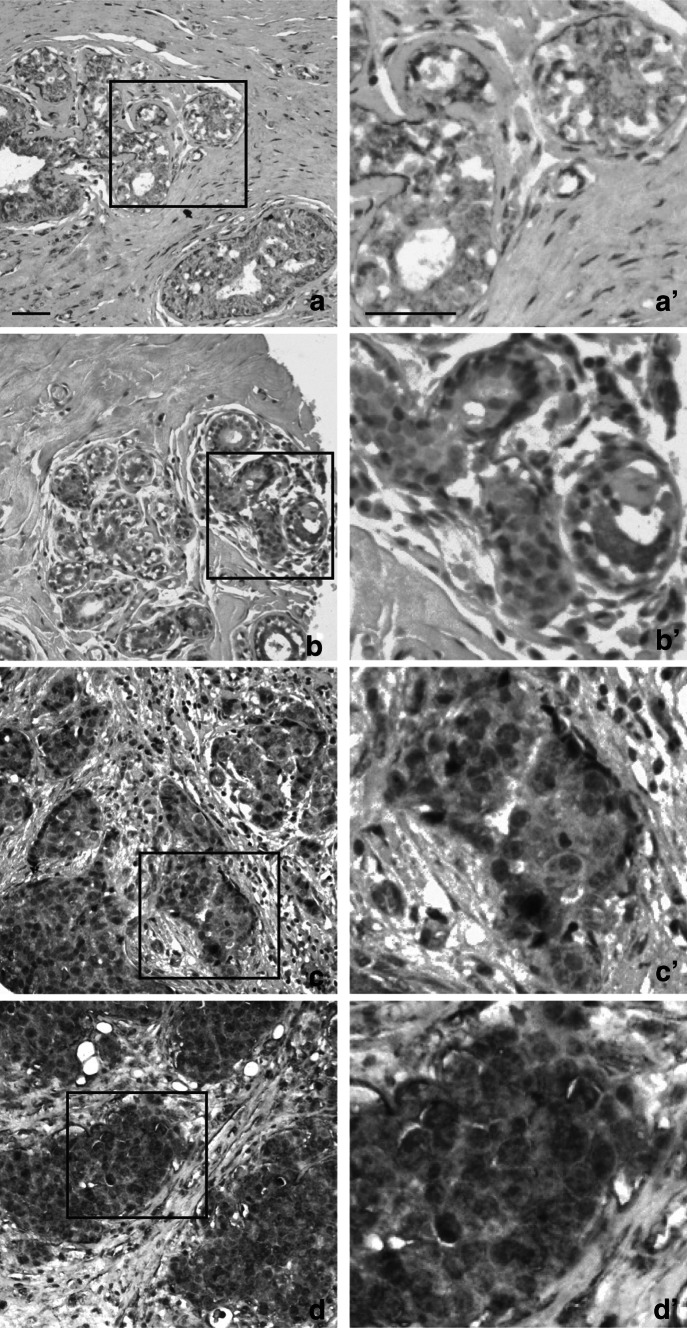
Immunohistochemistry detection of KGFR in breast cancer. Breast tissue array was subjected to immunohistochemistry with SC-101 mAb. **a**–**d** Representative tissue sections for each sample are shown (original magnification 20×, *scale bar* 50 μm). A significant area in each panel is indicated by a square and an enlargement of this area is shown in the respective **a′**–**d′** panels (original magnification 40×, *scale bar* 50 μm). Staining intensity was calculated for each category. Briefly, five patients for each group were processed and three images were taken from each patient. Mean values were obtained from five measurements of each image and classified as scores according to Table 2. Normal (panels **a**, **a′**) and NAT tissues (panels **b**, **b′**) showed weak positive staining (score 1), while grade 2 ductal breast cancer (panels **c**, **c′**) was classified as moderate positive (score 2) and grade 3 ductal breast cancer (panels **d**, **d′**) showed strong positive staining (score 3)

Since it has been proposed a role for KGFR in breast cancer [3], we analyzed SC-101 mAb ability to detect KGFR by IHC analysis employing a tissue array bearing 40 invasive ductal breast cancer samples (grade 2–3). In particular, we investigated the relationship between KGFR expression obtained with SC-101 mAb and tumor grade. As shown in Fig. S1, KGFR staining is higher in grade 2 and grade 3 of breast cancer tissue (Fig. S1, panel C and panel D, respectively) respect to normal tissue (Fig. S1, panel A). Instead we observed slightly more intense signal in normal adjacent tissue (NAT) (Fig. S1, panel B) respect to normal tissue. In particular, quantification of IHC signal (Table 5), obtained by multiplying the percentage of positive KGFR-staining area measured by means of ImageJ software and the staining intensity score, revealed a final IHC score of 20.5 ± 7.8 in normal breast tissue (Fig. 4, panels A, A′), which corresponded to a weak positive staining (score 1), according to our classification (Table 2). NAT showed a low score of specific staining for KGFR (26.5 ± 4.2, score 1) (Fig. 4, panels B, B′) as well. Conversely, a significantly higher IHC score was observed in grade 2 cancer tissue (107.9 ± 34.4, score 2) (*p* < 0.01 *vs* normal tissue) (Fig. 4, panels C, C′). Moreover, grade 3 breast cancer (Fig. 4, panels D, D′) showed even higher intensity than grade 2 (171.2 ± 39.3, score 3) (*p* < 0.05 *vs* grade 2).

**Table 5:** Mean values of staining intensity, percentage of positively stained area and final immunohistochemical score in breast samples group

	Positive area (%)	Intensity	IHC score	*p* value
Normal	21.9 ± 2.5	0.9 ± 0.3	20.5 ± 7.8	
NAT	26.5 ± 4.2	1 ± 0	26.5 ± 4.2	–
Grade 2	54.5 ± 2.2	2 ± 0.7	107.9 ± 34.4	*p* < 0.01
Grade 3	68.6 ± 4.6	2.5 ± 0.6	171.2 ± 39.3	*p* < 0.05

To confirm the reliability of SC-101 mAb in detecting KGFR expression, we assessed KGFR mRNA levels by Real-Time PCR on bioptic specimens of ductal breast cancer and normal breast tissue. Real-Time PCR confirmed at molecular level the overexpression of KGFR in cancer (2.5 fold, *p* < 0.05), while FGFR2-IIIc did not increase its expression in tumoral tissue (1.1-fold) (Fig. 7a).

### Immunohistochemical Detection of KGFR in Pancreatic Cancer

**Fig. 5:**
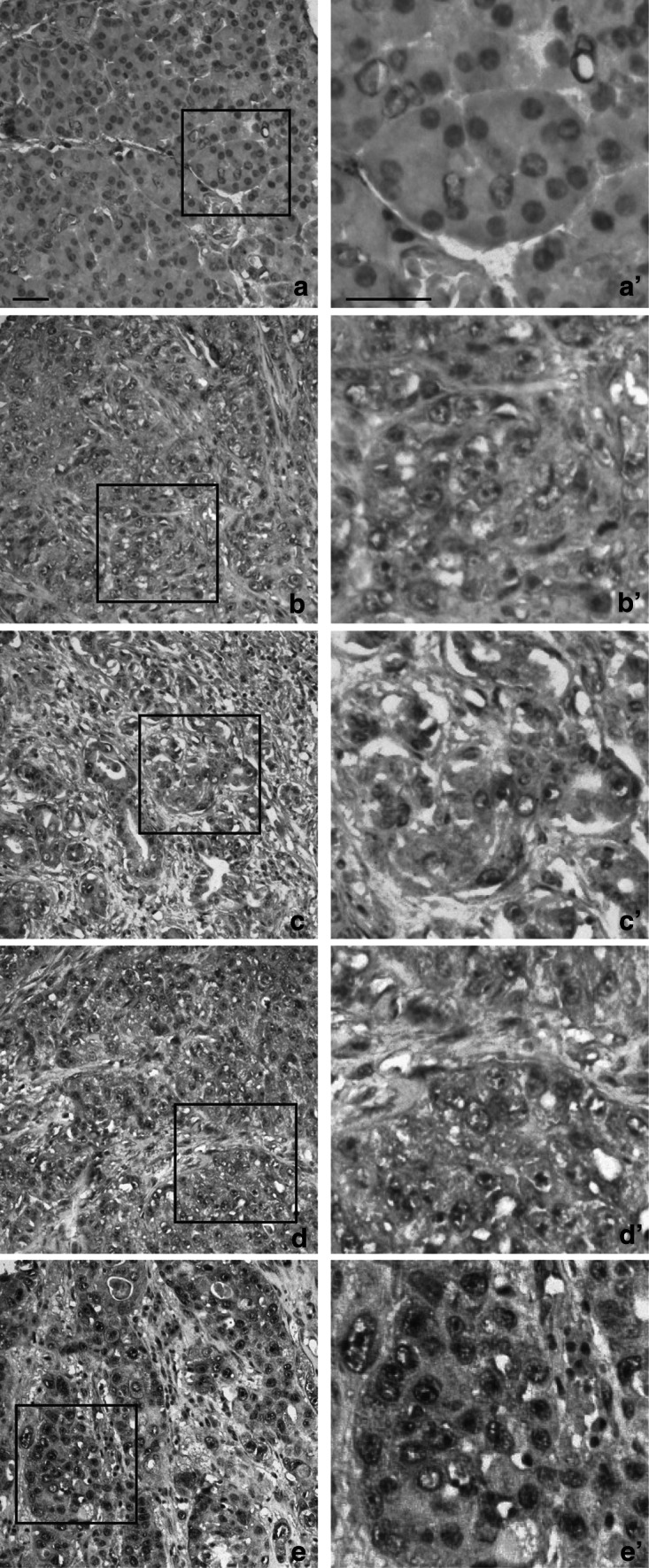
Immunohistochemistry detection of KGFR in pancreatic cancer. Pancreas tissue array was subjected to immunohistochemistry with SC-101 mAb. **a**–**e** Representative tissue sections for each sample are shown (original magnification 20×, *scale bar* 50 μm). A significant area in each panel is indicated by a square and an enlargement of this area is shown in the respective **a′-e′** panels (original magnification 40×, *scale bar* 50 μm). Staining intensity was calculated for each category. Briefly, five patients for each group were processed and three images were taken from each patient. Mean values were obtained from five measurements of each image and classified as scores according to Table 2. Normal (panels **a**, **a′**) and NAT tissues (panels **b**, **b′**) expressed weak positive staining (score 1), grade 1 (panels **c**, **c′**) and grade 2 duct adenocarcinoma (panels **d, d′**) were assigned a score 2 (moderate positive staining), while grade 3 duct adenocarcinoma (panels **e**, **e′**) showed a strong positive staining (score 3)

We also investigated KGFR expression by IHC-employing SC-101 mAb in pancreatic cancer, which is known to express high levels of this receptor [3]. We analyzed a tissue array of 40 cases of duct adenocarcinoma, grade 1–3. In pancreatic cancer, we observed high intensity of KGFR staining correlated to tumor grade (Fig. S2, panel C grade 1, panel D grade 2 and panel E grade 3) respect to normal tissue (Fig. S2, panel A). NAT shown slight increase in KGFR-staining intensity (Fig. S2, panel B) respect to normal tissue. In particular, a faint IHC score for KGFR (Table 6) was observed in normal tissue (18.4 ± 5.4, score 1) (Fig. 5, panels A, A′) and a slightly more intense signal was evident in NAT (34.7 ± 6.7, score 1) (Fig. 5, panels B, B′). SC-101 mAb showed a significant increase in IHC score in grade 1 and grade 2 tumor sections (78.7 ± 22.0 and 124.7 ± 39.1, respectively, score 2) (*p* < 0.01 and *p* < 0.01 *vs* normal tissue, respectively) (Fig. 5, panels C, C′ and D, D′). However, membrane and cytoplasmic KGFR staining became further more evident and reached its higher level in grade 3 specimens (210.0 ± 41.3, score 3) (*p* < 0.05 *vs* grade 2) (Fig. 5, panel E, E′).

**Table 6:** Mean values of staining intensity, percentage of positively stained area and final immunohistochemical score in pancreas samples group

	Positive area (%)	Intensity	IHC score	*p* value
Normal	16.0 ± 3.1	1.2 ± 0.4	18.4 ± 5.4	
NAT	34.7 ± 6.7	1 ± 0	34.7 ± 6.7	–
Grade 1	47.3 ± 2.8	1.6 ± 0.5	78.7 ± 22.0	*p* < 0.01
Grade 2	55.9 ± 5.5	2.2 ± 0.4	124.7 ± 39.1	*p* < 0.01
Grade 3	76.4 ± 5.4	2.8 ± 0.5	210.0 ± 41.3	*p* < 0.05

We performed Real-Time PCR analysis on mRNA obtained from biopsies of pancreatic adenocarcinoma (Fig. 7b). Molecular analysis confirmed KGFR overexpression in tumoral tissue (4.6–fold, *p* < 0.01). As concerning FGFR2-IIIc isoform, we observed an increase in its expression in cancer specimens, although less marked than that of KGFR (3.3-fold, *p* < 0.01).

### Immunohistochemical Detection of KGFR in Thyroid Cancer

**Fig. 6:**
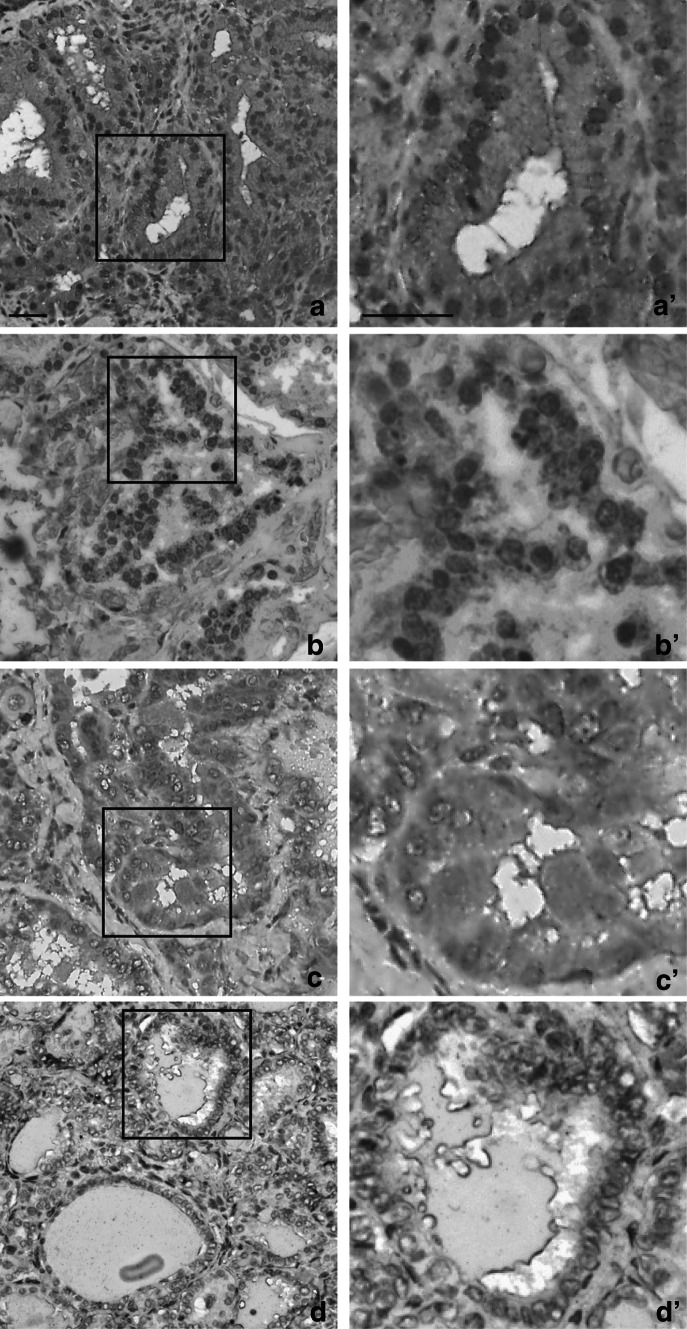
Immunohistochemistry detection of KGFR in thyroid cancer. Normal thyroid and thyroid papillary cancer specimens were subjected to immunohistochemistry with the commercial Bek antibody (panels **a, a′** and **c**, **c′**) or with SC-101 mAb (panels **b**, **b′** and **d**, **d′**). **a**–**d** Representative tissue sections for each sample are shown (original magnification 20×, *scale bar* 50 μm). A significant area in each panel is indicated by a *square* and an enlargement of this area is shown in the respective **a′**–**d′** panels (original magnification 40×, *scale bar* 50 μm). Staining intensity was calculated for each category. Briefly, five patients for each group were processed and three images were taken from each patient. Mean values were obtained from five measurements of each image and classified as scores according to Table 2. Bek antibody revealed a strong staining in normal tissue (panels **a**, **a′** score 3), with a partial reduction in cancer specimens (panels **c**, **c′** score 2). SC-101 mAb staining was classified as score 2 in normal thyroid tissue (panels **b**, **b′**) and score 0 in cancer samples (panels **d**, **d′**)

It has been previously demonstrated a significant FGFR2 down-modulation in thyroid cancer [4]. Nevertheless, we observed that IHC staining performed with the commercial Bek antibody (Table 7) could highlight only a partial decrease of FGFR2 IHC score in thyroid cancer samples (Fig. 6, panels C, C′), with respect to normal thyroid tissue (Fig. 6, panels A, A′) (145.6 ± 34.8, score 2, *vs* 220.6 ± 6.3, score 3), thus indicating that Bek antibody was not efficient to detect significant variations of FGFR2 expression in thyroid carcinoma due to poor sensitivity and high background.

**Table 7:** Mean values of staining intensity, percentage of positively stained area and final immunohistochemical score in thyroid samples group

		Positive area (%)	Intensity	IHC score	*p* value
SC-101	Normal	52.7 ± 1.4	2.7 ± 0.6	140 ± 27.4	
	Tumor	12.8 ± 2.4	0.8 ± 0.4	9.4 ± 5.3	*p* < 0.05
Bek C-17	Normal	73.5 ± 2.1	3.0 ± 0	220.6 ± 6.3	
	Tumor	58.6 ± 2.6	2.5 ± 0.7	145.6 ± 34.8	–

Since thyroid follicular cells involved in neoplastic transformation are essentially derived from epithelial lineage, we assumed that our KGFR-specific SC-101 mAb could be suitable to highlight FGFR2 modulation in this cancer. Indeed, SC-101 mAb, due to its high specificity and sensitivity, was able to detect a strong membrane staining in normal tissue, corresponding to an IHC score of 140 ± 27.4 (score 2) (Fig. 6, panels B, B′), and virtually no signal in papillary thyroid carcinoma samples, with an IHC score of 9.4 ± 5.3 (score 0) (*p* < 0.05 *vs* normal tissue) (Fig. 6, panels D, D′).

**Fig. 7:**
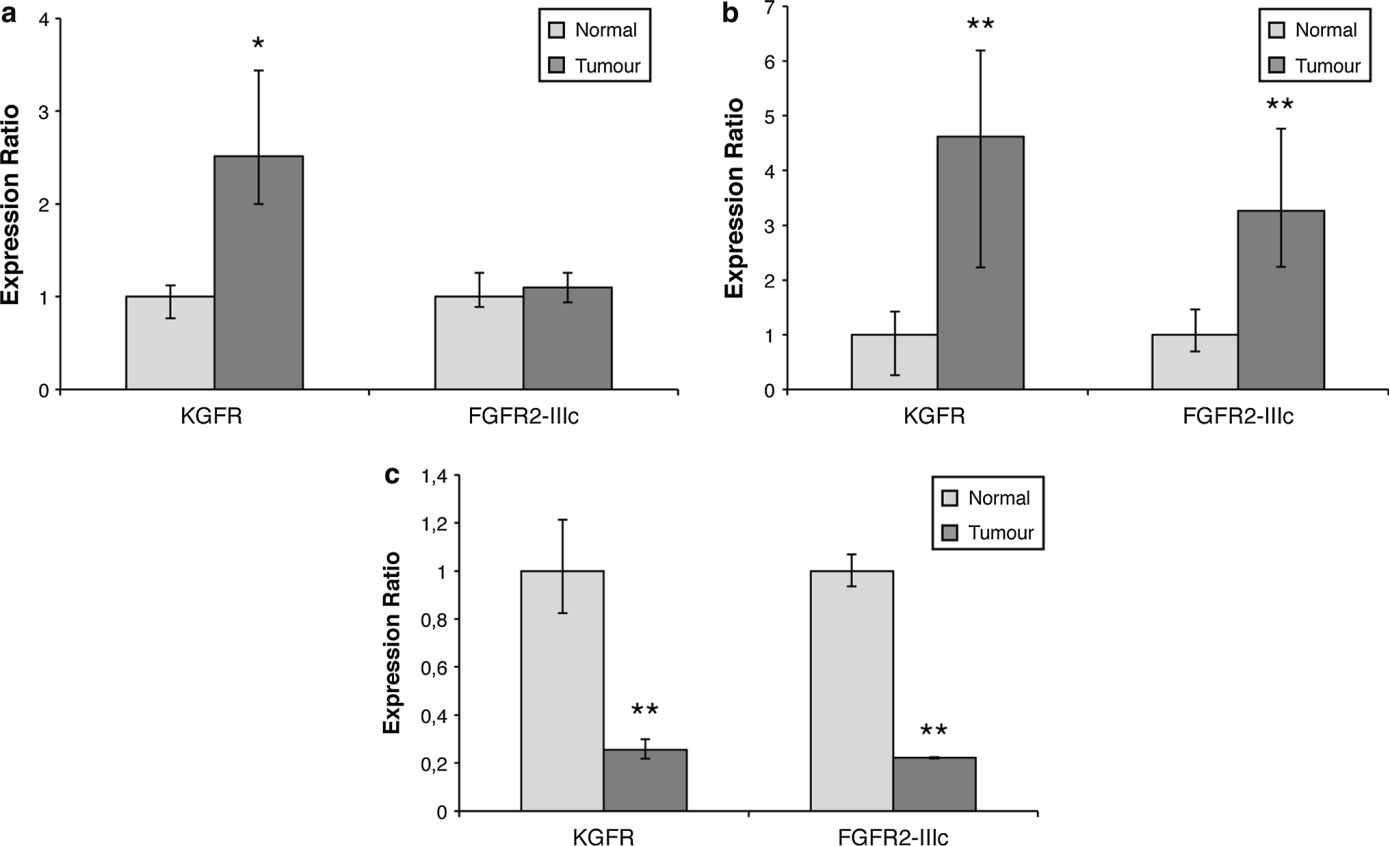
Real-Time PCR detection of KGFR in breast, pancreatic and thyroid cancer. **a** Quantitative Real-Time PCR analysis of KGFR and FGFR2-IIIc mRNA expression in breast cancer specimens. Relative KGFR or FGFR2-IIIc mRNA levels are shown as fold value of the level of the corresponding mRNA in normal breast tissue. *Error bars* represent standard deviations. *p < 0.05. **b** Quantitative Real-Time PCR analysis of KGFR and FGFR2-IIIc mRNA expression in pancreatic cancer specimens. Relative KGFR or FGFR2-IIIc mRNA levels are shown as fold value of the level of the corresponding mRNA in normal pancreatic tissue. *Error bars* represent standard deviations. **p < 0.01. **c** Quantitative Real-Time PCR analysis of KGFR and FGFR2-IIIc mRNA expression in thyroid cancer specimens. Relative KGFR or FGFR2-IIIc mRNA levels are shown as fold value of the level of the corresponding mRNA in normal thyroid tissue. *Error bars* represent standard deviations. **p < 0.01

KGFR expression was also assessed through Real-Time PCR on mRNA obtained from biopsies of papillary thyroid carcinoma and normal tissue. This analysis confirmed both KGFR and FGFR2-IIIc down-modulation (0.3-fold and 0.2-fold, respectively, *p* < 0.01) in thyroid carcinomas (Fig. 7c).

## Discussion

Growth factors and their tyrosine kinase receptors are involved in several cellular processes, such as proliferation, growth, differentiation, migration, and survival [13, 14]. These receptors play pivotal biological roles during the development and adult life of multicellular organisms, and their deregulation has been linked to the establishment of cancer. In this work, we focused on KGFR, a tyrosine kinase receptor generated by an alternative splicing of FGFR2 gene. Its activity is most often recognized as oncogenic, although in some cases KGFR has been shown to behave as a tumor suppressor. In particular, dysregulation of FGFR2 gene has been reported in thyroid, breast, lung, stomach, and pancreatic cancers, in which up- or down-modulation of KGFR expression has been observed in epithelial cells [3, 4, 8, 15], and in melanoma, where loss-of-function FGFR2 mutations have been detected in cancer specimens and cell lines [5]. Moreover, our previous works showed an increased KGFR expression also in Kaposi Sarcoma and in dermatofibroma, a benign skin tumor [16, 17]. However, KGFR detection by IHC in bioptic specimens is impaired by the lack of commercially available antibodies capable to discriminate between the two isoforms of the FGFR2 gene. Here, we suggest a potential diagnostic and prognostic strategy based on the introduction of a new monoclonal antibody specific for KGFR (named SC-101 mAb). To generate a KGFR-specific mAb, we amplified exon 8 sequence and used the corresponding peptide as an immunogen.

This antibody showed to be effective in several applications, such as Western blotting, immunofluorescence, and immunoprecipitation. Its specificity for KGFR isoform has been confirmed by Western blotting on human fibroblasts. Since commercially available antibodies do not discriminate between the two isoforms, and given the well-known involvement of KGFR in several human diseases, including cancer, the introduction of SC-101 mAb can be advantageous in clinical applications. Nowadays, expression levels of human epidermal growth factor receptor 2 (HER2) [18] and other proteins such as estrogen receptor (ER) [19] are routinely investigated by IHC in breast cancer diagnostic procedures. To assess a possible diagnostic use of SC-101 mAb, we analyzed its ability to detect KGFR by immunohistochemistry employing different epithelial tumors characterized by KGFR-altered expression. Here we demonstrated that SC-101 mAb was able to disclose KGFR overexpression in breast cancer samples compared to normal tissue. Furthermore, we assessed that the intensity of KGFR immunostaining was directly proportional to the tumor grade. Therefore, it is conceivable that the score of positivity to SC-101 mAb might have a significant prognostic value in breast cancer. A further advantage of a KGFR-specific antibody is represented by the fact that both FGFR2 isoforms are expressed in normal tissue, whereas tumor samples expressed mainly KGFR, as previously reported by the literature [20] and as also confirmed by our Real-Time PCR data (Fig. 7a).

We also investigated KGFR expression in pancreatic adenocarcinoma, which has been linked to KGFR overexpression [21]. Again, SC-101 mAb was able to detect KGFR overexpression in pancreatic adenocarcinoma and, also in this case, we could establish a positive correlation between tumor grade and protein expression.

In other tumors, FGFR2 has been suggested to exert tumor suppressor activity, as it happens in thyroid, prostate and bladder cancers [4, 22–24]. In particular, FGFR2 is the only member of FGFRs family to be consistently expressed in normal human thyroid tissue and strongly down-modulated in thyroid cancer [4]. Once again, SC-101 mAb was able to detect strong KGFR expression levels in normal thyroid tissue and very low KGFR staining in thyroid carcinoma samples, while with the commercially available Bek antibody we obtained a higher background and a positive signal also in tumor specimens. Therefore, this antibody seems to be sensitive enough to highlight KGFR down-modulation occurring in thyroid carcinoma.

Previous works just described the production of KGFR-specific monoclonal antibodies aimed to neutralize its ligand-dependent activation for potential therapeutic use [25, 26], as already exist for epidermal growth factor receptor (EGFR) [27, 28]. Nevertheless, at our knowledge, no KGFR-specific antibodies have been proposed as diagnostic and prognostic tools until now.

## Conclusions

In this work, we demonstrated that SC-101 mAb is able to selectively detect KGFR isoform, and it works in the most common research applications (Western blotting, immunoprecipitation, immunofluorescence, IHC), giving better results in comparison to the commercially available Bek antibody in terms of background reduction, sensitivity at lower concentrations and specificity for KGFR isoform, thus indicating its suitability in basic research. Moreover, SC-101 mAb also showed a good potential as a diagnostic tool. Several proteins, such as ER and EGFR, are currently evaluated in cancer diagnosis, and the correlation between their expression and tumor stage, differentiation degree or aggressiveness led also to their use in prognostic applications. Interestingly, we observed a significant difference in KGFR distribution in different cancer grades. In fact, IHC analysis displayed an increase in KGFR staining that is directly proportional to the tumor grade. Thus, KGFR expression appears to be correlated to cancer progression.

Overall, we believe that SC-101 mAb, which specifically detects KGFR isoform, might represent a useful tool for use in basic research and it could also improve the accuracy of diagnosis and prognosis of epithelial tumors characterized by a modulation of KGFR expression.

## Electronic supplementary material

Below is the link to the electronic supplementary material.

Figure S1. Immunohistochemistry detection of KGFR in breast cancer. Breast tissues were subjected to immunohistochemistry with SC-101 mAb. Representative tissue sections for each sample are shown (original magnification 20x, scale bar 50 μm). Normal and NAT tissues (panels A and B, respectively), grade 2 ductal breast cancer (panel C) and grade 3 ductal breast cancer (panel D). (TIFF 3211 kb)

Figure S2. Immunohistochemistry detection of KGFR in pancreatic cancer. Pancreas tissues were subjected to immunohistochemistry with SC-101 mAb. Representative tissue sections for each sample are shown (original magnification 20x, scale bar 50 μm). Normal and NAT tissues (panels A and B, respectively), grade 1 and grade 2 duct adenocarcinoma (panels C and D, respectively) and grade 3 duct adenocarcinoma (panel E). (TIFF 3708 kb)
