# Supplementary figures and images for: Potential Prognostic and Diagnostic Application of a Novel Monoclonal Antibody Against Keratinocyte Growth Factor Receptor
Source: Mol Biotechnol. 2014 Jun 5;56(10):939–52. doi: 10.1007/s12033-014-9773-x (PMC4155171; doi:10.1007/s12033-014-9773-x)

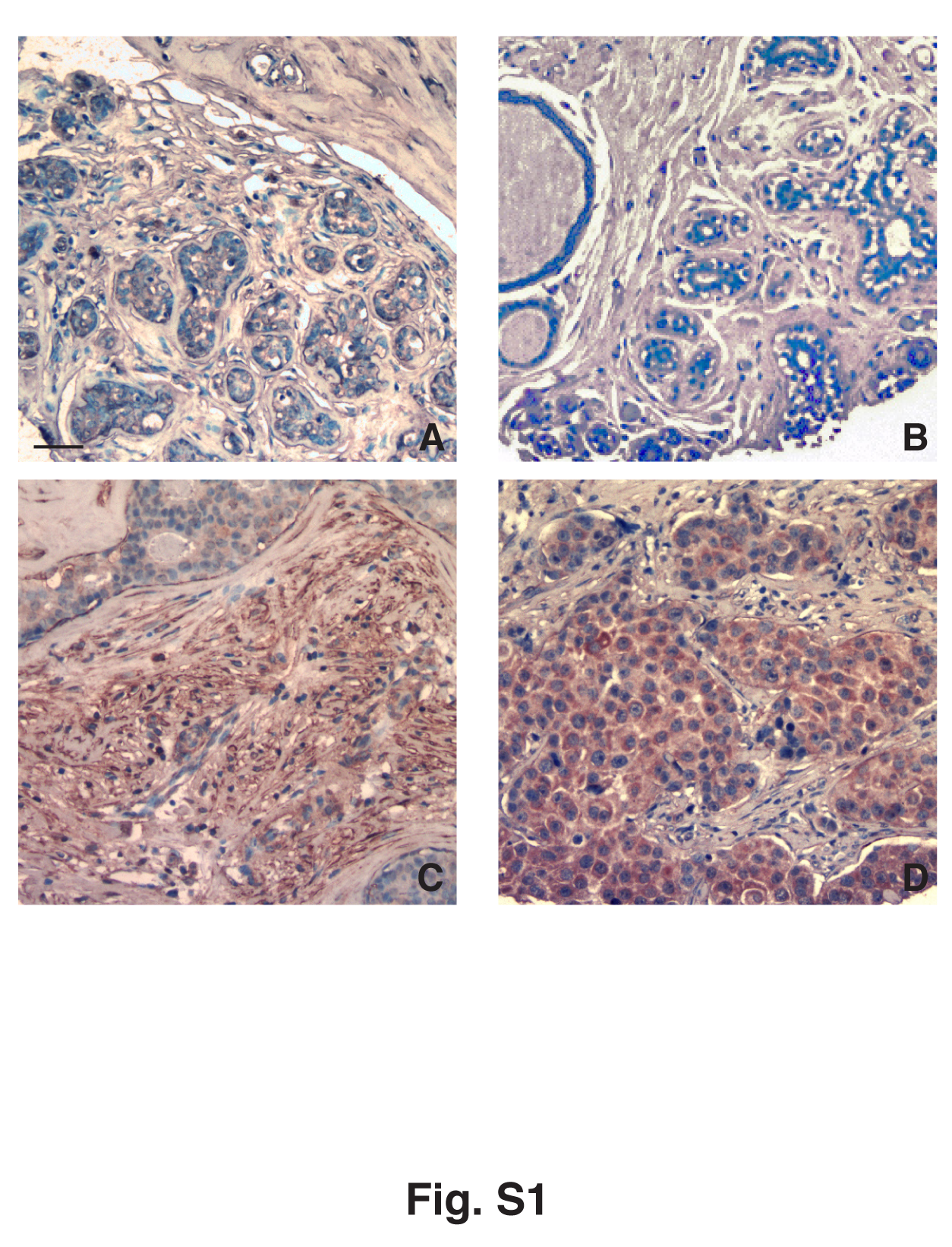

Supplement: Supplementary file 1 — Figure S1. Immunohistochemistry detection of KGFR in breast cancer. Breast tissues were subjected to immunohistochemistry with SC-101 mAb. Representative tissue sections for each sample are shown (original magnification 20x, scale bar 50 μm). Normal and NAT tissues (panels A and B, respectively), grade 2 ductal breast cancer (panel C) and grade 3 ductal breast cancer (panel D). (TIFF 3211 kb) [file 12033_2014_9773_MOESM1_ESM.tif]

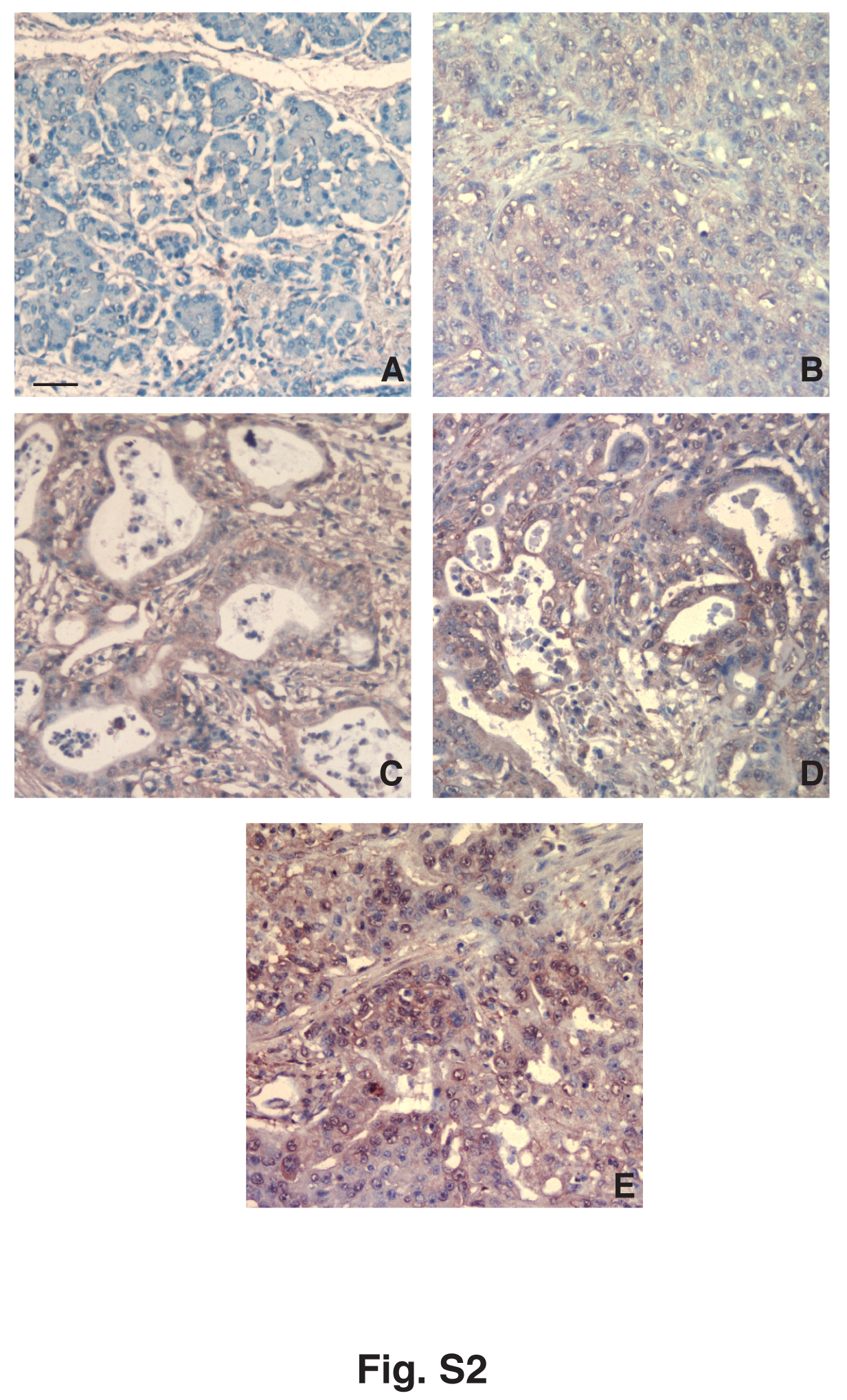

Supplement: Supplementary file 2 — Figure S2. Immunohistochemistry detection of KGFR in pancreatic cancer. Pancreas tissues were subjected to immunohistochemistry with SC-101 mAb. Representative tissue sections for each sample are shown (original magnification 20x, scale bar 50 μm). Normal and NAT tissues (panels A and B, respectively), grade 1 and grade 2 duct adenocarcinoma (panels C and D, respectively) and grade 3 duct adenocarcinoma (panel E). (TIFF 3708 kb) [file 12033_2014_9773_MOESM2_ESM.tif]
